# Multiphoton Neurophotonics: Recent Advances in Imaging and Manipulating Neuronal Circuits

**DOI:** 10.1021/acsphotonics.4c02101

**Published:** 2025-04-04

**Authors:** Cécile Telliez, Ruth Sims, Giulia Faini, Pascal Berto, Eirini Papagiakoumou, Dimitrii Tanese, Nicolò Accanto

**Affiliations:** † 195517 Sorbonne Université, INSERM, CNRS, Institut de la Vision, Paris F-75012, France; ‡ 26907 Université Paris Cité, Paris 75006, France; § Institut Universitaire de France (IUF), Paris 75231, France; ∥ Institute for Bioengineering of Catalonia (IBEC), Barcelona Institute of Science and Technology, 08028 Barcelona, Spain

**Keywords:** Neurophotonics, Multiphoton microscopy, Optogenetic photostimulation, Calcium and voltage imaging, Wavefront shaping, All-optical brain studies

## Abstract

The possibility of using light to image and manipulate neuronal activity, at the heart of Neurophotonics, has provided new irreplaceable tools to study brain function. In particular, the combination of multiphoton microscopy and optogenetics allows researchers to interact with neuronal circuits with single-cell resolution in living brain tissues. However, significant optical challenges remain to empower new discoveries in Neuroscience. This Review focuses on three critical areas for future development: (1) expanding imaging and optogenetic stimulation to larger fields of view and faster acquisition speeds, while maintaining single-cell resolution and minimizing photodamage; (2) enabling access to deeper brain regions to study currently inaccessible neuronal circuits; and (3) developing optical techniques for studying natural behaviors in freely moving animals. For each of these challenges, we review the current state-of-the-art and suggest future directions with the potential to transform the field.

## Graphical abstract

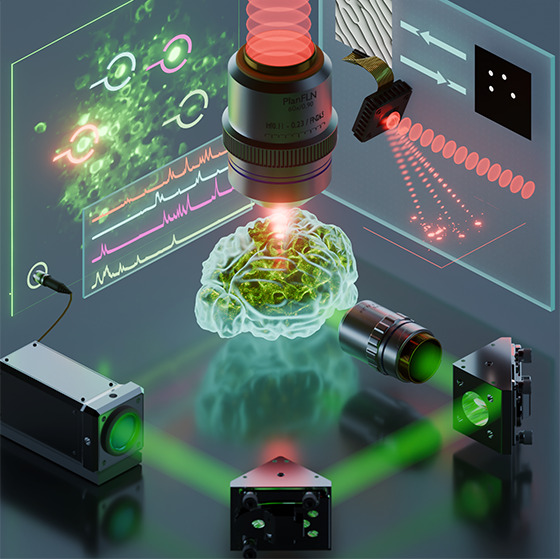

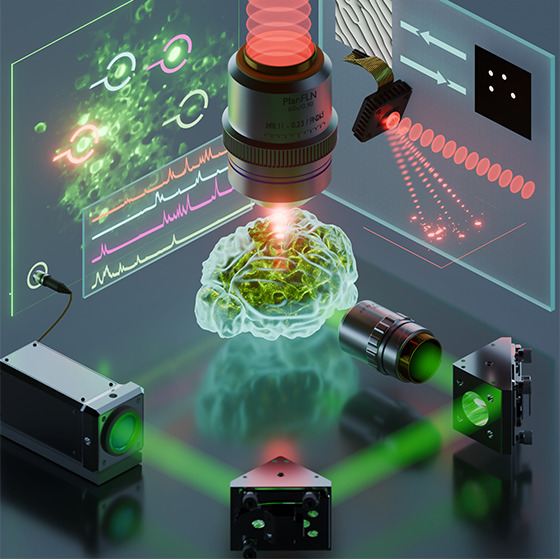

## Introduction

### Functional Imaging and Optogenetics

Neuronal circuits process information from the external world to shape our perception, memories, behavior, and ultimately, our identities. Understanding how exactly this processing takes place is a fundamental question in Neuroscience. Over the past 20 years, several game changing neurophotonic technologies have emerged, which address this challenge by providing powerful optical means of interacting with neuronal networks, empowered by two significant breakthroughs in genetic engineering. First, Genetically Encoded Calcium and Voltage Indicators (GECIs and GEVIs), are fluorescent proteins that, once expressed in neurons, enable imaging of neuronal function by converting changes in calcium ion concentration (a proxy for neuronal activity; in the case of GECIs) or changes in cell membrane potential (directly reflecting neuronal activity; in the case of GEVIs) into fluorescence variations.^1–4^ Second, the optogenetics technology relies on genetically expressing light-sensitive proteins, known as opsins, in specific neuronal populations, that enable researchers to control activity in those cells with light.^5–8^ By illuminating opsin-expressing neurons, it is possible to reversibly switch their activity on or off at will, in function of the type of opsin used.

While imaging neuronal activity establishes correlations - such as which neurons fire in correlation with a specific external stimulus - introducing controlled perturbations by selectively activating or inhibiting neuronal activity enables the inference of causal links,^9,10^ such as whether a specific group of neurons drives certain behaviors. Experiments in which neuronal activity imaging and optogenetics photostimulation can be performed at the same time are named “all-optical” studies of the brain.^11,12^ In the past 15 years, they have been used for instance for mapping functional connectivity among neurons^13–15^ and elucidating the neural bases of perception^16–20^ and behavior^14,19–23^ in small living animal models (mostly rodents).

Although one photon (1P) illumination methods (with visible light) for all-optical experiments have the advantage of simplicity and cost-effectiveness, and have been utilized in multiple studies,^24–29^ they are in general limited by background fluorescence, poor axial confinement, and limited propagation (<200 μm) through scattering tissues such as the brain. Consequently, most all-optical studies in mice, a widely used animal model, use Two-Photon Laser Scanning Microscopy (2P-LSM) for its superior tissue penetration and axial confinement.^30–32^ In this manuscript we will mainly focus on 2P studies of the brain, both for functional imaging and optogenetic photostimulation, while giving the relevant references for the newest 1P approaches and detailing the potential of 3P microscopy for reaching deeper brain regions.

### Two-Photon All-Optical Studies of the Brain

2P-LSM is today the gold standard for imaging neurons and neuronal activity *in vivo* in the mouse brain. By far, the most used GECI is the GCaMP family,^1–3,33–35^ whose absorption peak in the 2P regime sits around 920 nm. To construct an image (Figure 1a,b), a near-infrared (NIR) pulsed laser centered at 920 nm (typically Ti:sapphire oscillators with <200 fs pulse duration, 80 MHz repetition rate and tens of nJ pulse energy) is focused by a microscope objective on a diffraction-limited spot that is raster scanned by a pair of galvanometric mirrors across the field of view (FOV). The fluorescence photons emitted by the activity indicator, are collected through the same objective and detected with a photomultiplier tube (PMT).

**Figure 1:**
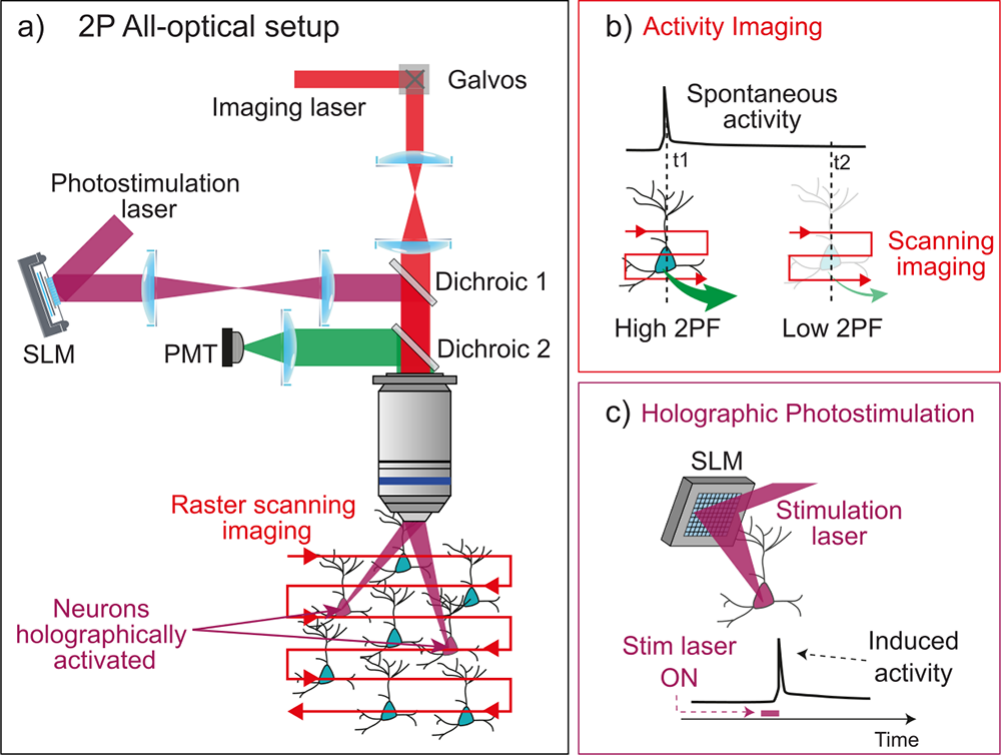
Two-photon all-optical studies of the brain. (a) Setup for 2P all-optical studies of the brain, composed of an imaging path (red) to record the activity of neurons within a network and a photostimulation path (purple) to induce controlled optogenetic perturbations (here, activation of neurons). In the imaging path, a near-infrared (NIR) laser is raster-scanned across the field of view and the 2P fluorescence is recorded with a photomultiplier tube (PMT). Optogenetic stimulation is induced by shaping the light from a second NIR laser with a spatial light modulator (SLM), which redirects the light to precisely target the neurons of interest. (b) Simplified sketch of the 2P activity imaging process. Upon raster scanning of the imaging laser on the field of view, neurons expressing a fluorescent activity reporter emit a 2P Fluorescence (2PF) signal, with its intensity reflecting the level of spontaneous activity of the neuron. (c) Simplified sketch of the 2P patterned optogenetic photostimulation processes. Upon brief patterned illumination from the SLM, a targeted neuron expressing an optogenetic actuator (or inhibitor; not shown) has its activity induced (or suppressed).

In a 2P all-optical experiment (Figure 1a,c), a second NIR pulsed laser is used for optogenetic photostimulation. When using GCaMP as the activity indicator, the typical choice is to use red-shifted opsins^13,16,19,21^ that can be efficiently excited at near-infrared wavelengths. This has the great advantage of meeting the high energy demands of optogenetic activation based on powerful laser amplifiers using Ytterbium-doped active media, which have seen significant industrial development in recent years, are centered around 1030–1070 nm, and can deliver ~300 fs laser pulses with low repetition rates (0.5–10 MHz) and very high energy (>50 μJ). However, as most microbial opsins can also be excited at 920 nm, this configuration is prone to generate physiological cross-talk, i.e. spurious activation of opsin-expressing neurons by the imaging laser. Strategies to minimize the cross-talk include:^36^ limiting the imaging laser power, imaging across larger FOVs to limit the dwell time per pixel, or switching to a configuration based on red-shifted GECIs and blue-shifted opsins^37,38^ that effectively minimizes the spectral overlap.

To selectively photostimulate only the neurons of interest among those expressing an opsin, 2P stimulation is combined with light shaping techniques to split the laser beam into tens of spots that simultaneously illuminate target neurons (Figure 1a). A preferred light shaping method is Computer-Generated Holography (CGH),^21,39–41^ implemented using a liquid crystal Spatial Light Modulator (SLM) (Figure 1a,c). The SLM can either create multiple diffraction-limited spots that are spirally scanned with galvanometric mirrors to illuminate the entire cell body (soma) of neurons,^42,43^ or directly sculpt extended shapes (10–15 μm disks) to target the soma in a scanless manner. In 2P holographic optogenetics, all targets are therefore illuminated simultaneously and we will thus refer to this holographic 2P photostimulation process as “parallel” excitation, as opposed to the serial nature of scanning excitation used for imaging.

### Manuscript Outline

As all-optical studies begin to advance our understanding of the brain, major optical challenges must be addressed. This article focuses on three key challenges (illustrated in Figure 2), briefly reviews the current state of the art and outlines future directions that could significantly advance Neurophotonics.

**Figure 2:**
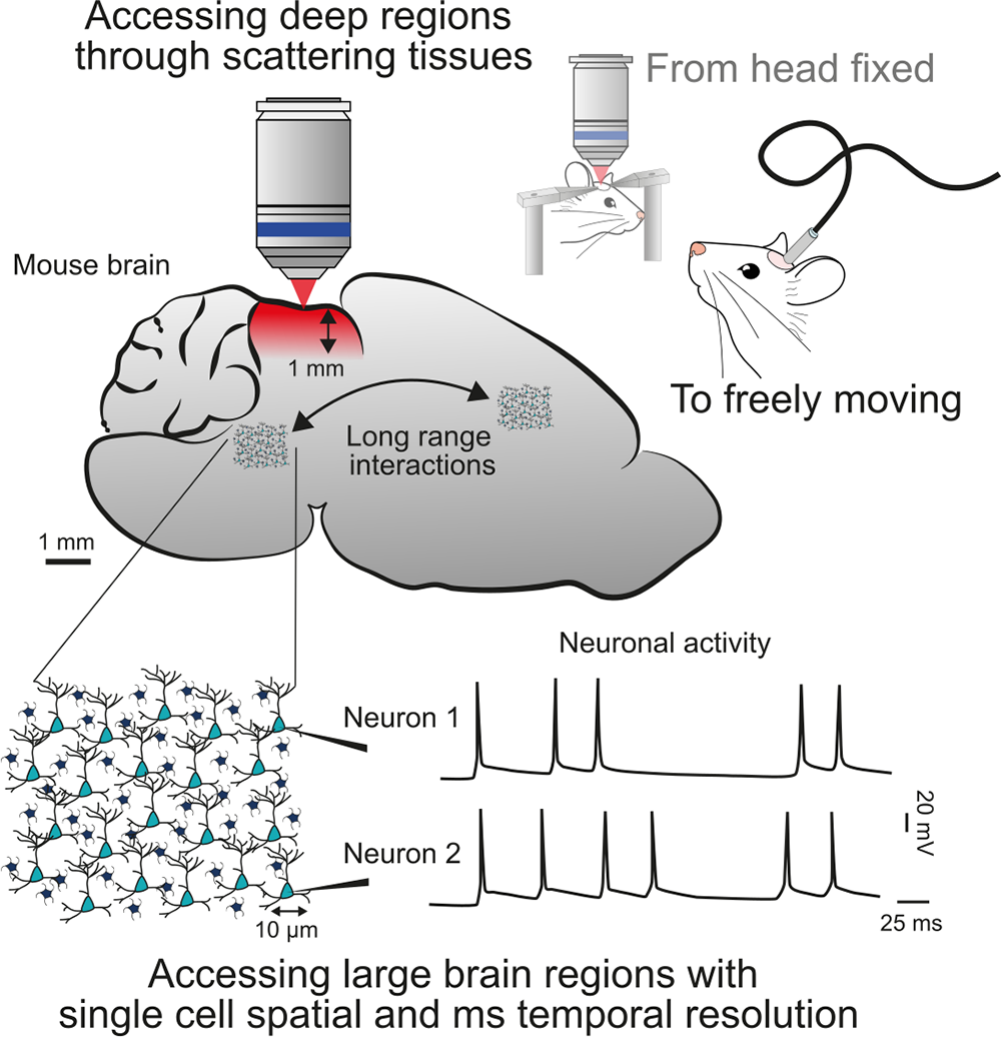
Current challenges in all-optical brain interrogation. Current challenges in all-optical brain interrogation include: (1) imaging deep, down to a mm, within brain tissue, where scattering and absorption limit light penetration and signal quality; (2) adapting optical systems to freely moving animals, requiring miniaturization of microscopes and stable imaging and stimulation; and (3) achieving larger-scale, high-speed, and accurate imaging and stimulation of neuronal networks across broader brain regions.

Part A explores methods for imaging large neuronal networks with high spatial resolution and speed. We discuss strategies for expanding the FOV in 2P imaging and photostimulation, achieving kHz imaging speeds for voltage indicators, and enabling ultrafast optogenetic activation of targeted neurons. We conclude with a discussion on limitations related to the tissue damage and strategies to scale up circuit investigations without exceeding photodamage threshold.

Part B focuses on the challenge of reaching deeper brain regions through scattering tissue. Depending on the depth, solutions include using longer excitation wavelengths, as in three-photon (3P) microscopy, adaptive optics and scattering compensation techniques, and microendoscopy.

Part C highlights advances in flexible and adaptable technologies designed to study natural behavior in freely moving animals.

## Part A. Precisely Probing Neuronal Circuits Across Millimeter Fields of View and at Kilohertz Rates

In an ideal scenario, all-optical experiments would grant imaging and photostimulation access to an entire ensemble of neurons, with single-cell spatial resolution and at sufficient time scales for recording the time-dependent neuronal information without altering the brain tissue under study.

### Space

Neuronal circuits, from local to large-scale networks, involve thousands to millions of neurons^44^ connected across distant brain regions,^45^ dynamically processing and integrating information. As a consequence, focusing solely on a single region or a subset of neurons may not fully capture how neurons encode information.^45,46^ To address this, all-optical microscopy techniques must be developed to record and manipulate the activity of multiple, large (∼mm) brain areas involved in processing the information under study, while maintaining at least single-cell resolution. It is important to emphasize, however, that the performance of an all-optical setup is not solely determined by the optics. In the case of single-cell optogenetic photostimulation, for example, the spatial resolution also depends on the expression pattern of the opsins. Neuronal cell bodies are often densely surrounded by neurites of neighboring cells, and if opsins are expressed in these neurites, even 2P stimulation targeted to a single cell body can inadvertently activate dendrites or axons of nearby neurons, effectively reducing spatial resolution. To overcome this issue, soma-targeted opsins have been developed, which confine opsin expression to the cell body while excluding it from neuronal processes, thereby enhancing the spatial precision of photostimulation.^47–49^

### Time

In neuronal computation, information is electrically encoded through changes in membrane potential. Smaller variation of this potential, below a certain threshold and defined as subthreshold fluctuations, serve as graded analogue signal within neurons, while supra-threshold changes trigger Action Potentials (APs) – brief (few ms) electrical spikes that propagate to downstream neurons. Neurons fire APs at precise times (on the ms scale) and specific frequencies (from a few Hz to several hundreds of Hz), with some neurons firing in synchrony while others do not. Additionally, in some brain areas, e.g., superficial cortical layers, neuronal activity is generally sparse in time, with activation being mostly asynchronous and infrequent, with many neurons remaining silent for extended periods.^50–53^ All these temporal dynamics might be crucial for encoding sensation, cognition, and action in neural circuits.^54–56^ Whether information is encoded in the firing rate, the precise spike time or a combination of both,^57^ being able to optogenetically replicate naturally occurring spatiotemporal activity patterns, or interfere with them with high temporal accuracy is of fundamental importance for a better understanding of the neuronal code. From the imaging point of view, the kinetics of the activity reporter (calcium or voltage indicator) filter the dynamics of neuronal events and dictate the necessary acquisition frame rates to faithfully detect reported events. Calcium indicators capture changes in calcium concentration linked to AP generation and can be imaged effectively at frame rates from a few Hz to tens of Hz. In contrast, the faster and emerging voltage indicators require kHz frame rates to report both APs as well as subthreshold membrane potential changes.

### Balancing Spatial and Temporal Resolution

In sequential processes like typical 2P-LSM, one inherently has to accept trades off between the number of recorded positions, imaging speed and Signal-to-Noise Ratio (SNR). Striking a balance is crucial to maintain temporal resolution sufficient for capturing relevant activity events, while maximizing the number of recorded neurons and maintaining sufficient SNR to faithfully report activity. This necessitates strategic compromises to balance coverage, detail, imaging speed, and minimizing photodamage. In contrast, holographic optogenetics splits the laser beam to illuminate multiple neurons in parallel by using a SLM, effectively decoupling space and time. While previous reviews have already covered some of these trades off, especially from the point of view of imaging,^58–63^ this section will focus on unsolved challenges that have to be overcome to expand the FOV and the speed of functional imaging (calcium or voltage) and optogenetic photostimulation, while keeping light-induced damage of the sample within acceptable limits.

### Large-Scale Calcium Imaging

In the past years, different groups have developed large FOV^59,64–68^ (up to ∼5 mm in diameter) 2P microscopes (also called 2P mesoscopes) for calcium imaging with single neuron spatial resolution. These mesoscopes (see Figure 3a), based on custom designed lenses, carefully assembled scan engines made of different scanning mirrors in cascade, and microscope objectives with low aberrations, large pupils and wide accepting angles, can produce either faster acquisition (∼50 Hz) on multiple smaller regions across the whole 5 mm FOV (as in refs 66, 67 ), or slower acquisition speeds (∼1 Hz) of the entire FOV,^64^ with the possibility to acquire multiple planes at the same time,^65,68^ thus leading to the quasi simultaneous calcium imaging of up to ∼1 million neurons.^46,65^

**Figure 3:**
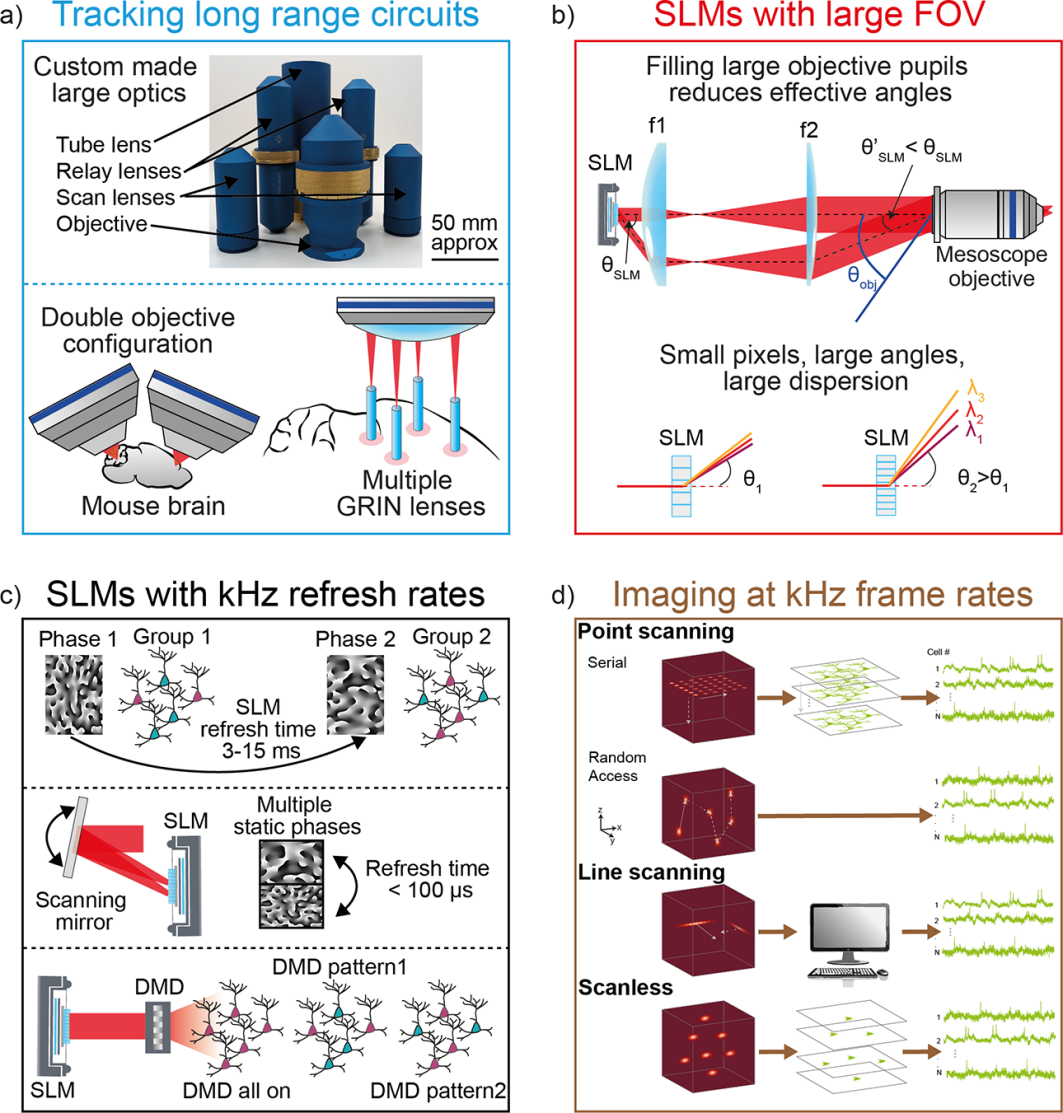
Strategies for fast and large 2P all-optical systems. (a) Large-Area Neuronal Activity Recording: neuronal activity across large regions can be recorded using custom-made large-field objectives and scanners (courtesy of Pacific Optica under the license https://creativecommons.org/licenses/by/4.0/, see also ref 67). Multiple areas can be accessed simultaneously by combining two objectives (as in^71,72^ ) or using multiple GRIN lenses.^73^ (b) Limitations that constrain the FOV accessible to a SLM positioned in the conjugate plane of the objective pupil, with the assumption that the objective pupil is fully illuminated. Top panel: filling the large pupil of mesoscope objectives (diameter > 30 mm) when using standard SLMs does not reach the acceptance angles of these objectives. Bottom panel: reducing the size of the SLM pixels increases the maximum achievable diffraction angles but also introduces significant chromatic dispersion. In 2P optogenetics this reduces the 2P photostimulation efficiency for spots generated off-center. (c) SLMs with kHz refresh rates. In holographic optogenetics, the refresh rate of liquid crystal SLMs is relatively slow (3–15 ms) and limits the maximum rate at which holograms can be projected and thus patterns reconfigured. To overcome this, fast (<100 μs) addressing of a multihologram SLM with galvanometric mirrors has been developed.^89^ Another approach combines a slow SLM with a fast DMD, which act as a rapid (10 kHz) area-specific shutter of the SLM-defined patterns.^90^ (d) State-of-the-art approaches for 2P imaging at kHz frame rates include serial scanning and scanless techniques. Simulated excitation point spread functions are shown on the left panels, with extracted single-cell activity traces on the right. Serial scanning methods can be divided into point-scanning, random access methods,^91,92^ and line-scanning tomography.^93^ Point-scanning uses a single diffraction-limited spot to image a volume via lateral and axial displacement, with fluorescence detected by PMTs. Random access methods scan targeted pixel sets of interest with acousto-optic deflectors,^94,95^ while line-scanning acquires angular projection of the FOV, and single-cell neuronal activity is recovered computationally. Scanless methods simultaneously illuminate several ROIs (using temporally focused, low-NA Gaussian, holographic or phase-contrast shaped beams^96^) and use camera detection to preserve spatial cell information.

One of the challenges associated with extremely powerful 2P mesoscopes is their current limited availability, largely confined to a few selected laboratories. While commercial versions of the 2P mesoscope of ref.^64^ (Thorlabs) or of ref 67 (Pacific Optica), are emerging, widespread adoption within neuroscience laboratories remains a crucial goal for the coming years. To this end, solutions that sacrifice some optical performance but within an easier and more cost-effective implementation could facilitate broader dissemination, especially when based on off-the-shelf components (as in ref. 69,70 ).

Pushing even beyond the spatial scales attainable with a 2P mesoscope, some research groups have explored techniques for parallel or sequential study of spatially separated regions, even centimeters apart.^71–74^ Parallelized systems (Figure 3a) have been developed using either dual beam paths with separate microscope objectives^71,72^ or multiple millimeter-sized lenses (GRIN lenses) coupled to a single objective.^73^ While the former offers superior resolution and detection efficiency, it is limited to simultaneous imaging of only two distant areas. The latter option, although theoretically more flexible, still requires complex alignment procedures. Inscopix recently introduced a 1P microscope variant, the Quartet system, employing four optical fibers, enabling more straightforward imaging of up to four different brain regions in mice. Expanding similar techniques, based on optical fibers, into the 2P domain holds promise for future advancements.

Finally, 1P widefield microscopes with camera detection inherently enable snapshot imaging, achieving millimeter (or even centimeter) scale FOVs and frame rates of tens of Hz.^75–78^ However, the large background fluorescence, limited axial confinement and penetration depth of 1P techniques make it difficult to identify real individual neuron activity. Investing in the further development of computational methods to demix fluorescence traces,^79–82^ as well as developing new smart approaches that combine structured illumination and adaptive optics,^83^ could be a promising direction. Computational innovations are also boosting 1P Light Field Microscopy,^84,85^ which further expands mesoscale access by enabling scanless 3D imaging at tens of Hz- by supporting its scalability, enabling near single-cell signal extraction, and extending depth access to a few hundreds of microns.

### Large-Scale Holographic Optogenetic Photostimulation

If the FOV achievable by a conventional 2P microscope has recently been expanded to the 5 × 5 mm^2^ limit,^64,67,86^ the laterally accessible FOV of a holographic 2P setup has, until now, been constrained to approximately 1 × 1 mm^2^ due to the inherent limitations of standard SLMs (see refs 16, 87 ). The most commonly used SLMs for 2P phase-modulation consist of a 2D matrix of ∼ 10^6^ liquid-crystal pixels. In a standard CGH configuration, the SLM is placed in the conjugate plane of the microscope objective pupil (see Figure 3b). Two relay lenses of focal lengths *f*_1_ and *f*_2_ are used to image the SLM screen at the objective pupil and to completely fill it. Let *L*_SLM_ be the lateral SLM screen size, the maximum holographic FOV at the sample plane (*L*_FOV_) can be expressed as follows:^88^

1 $$L_{\mathrm{FOV}}=2\frac{f_{\mathrm{obj}}}{M}\tan\left(\theta_{\mathrm{SLM}}\right)=\frac{D_{\mathrm{SLM}}}{{\mathrm{NA}}_{\mathrm{obj}}}\tan\left(\theta_{\mathrm{SLM}}\right)=\frac{\lambda N_{\mathrm{pix}}}{2{\mathrm{NA}}_{\mathrm{obj}}}$$

where *f*_obj_ and NA_obj_ are the focal length and numerical aperture (NA) of the objective, that can be related to the objective pupil diameter (*D*_Pup_) by the relation *D*_Pup_ = 2 NA_obj_ *f*_obj_; $M=\frac{f_{2}}{f_{1}}\approx \frac{D_{\mathrm{Pup}}}{L_{\mathrm{SLM}}}$ is the magnification between the SLM and pupil plane; θ_SLM_ is the maximal half angle that the SLM can introduce, which depends on the laser wavelength (λ) and the SLM pixel size (*d*_pix_): $\tan\left(\theta_{\mathrm{SLM}}\right)=\frac{\lambda }{2d_{\mathrm{pix}}}$ . The full SLM angular steering capabilities extends to ±θ_SLM_. *N*_pix_ is the number of SLM pixels in a line *L*_SLM_ = *N*_pix_ *d*_pix_. For typical commercial SLMs, screen sizes are around 15 mm, pixel sizes are 8–20 μm, giving a full angular coverage of about ±3.7 and ±1.5° at λ ≈ 1 μm. In comparison, 2P imaging mesoscopes with 5 × 5 mm^2^ FOV use customized objectives (Figure 3a) with NA_obj_ = 0.5, a very large pupil, *D*_Pup_ > 30 mm, and large acceptance angles θ_obj_ = ±5°. As schematically shown in Figure 3b, because of the magnification needed to fill the objective pupil, the SLM angles that are already intrinsically smaller than mesoscope acceptance angles, become even smaller at the objective entrance θ′_SLM_ < θ_SLM_ < θ_obj_. It follows that, coupling a standard SLM with a mesoscope objective, while filling its pupil results in a holographically accessible FOV of ∼1 × 1 mm^2^, as demonstrated in ref 87, which is much smaller than the FOV accessible for imaging. For comparison, scanning mirrors used in 2P imaging mesoscopes, have a size of 5–20 mm and an optical scanning angle of ∼±20°.

To develop a 2P holographic mesoscope, as derived from eq 1, one must increase the total number of SLM pixels. This can be done in two ways: (1) by decreasing the pixel size *d*_pix_ while keeping the SLM size *L*_SLM_ constant, which increases the steering angle, or (2) by increasing the SLM size while keeping the pixel size constant, reducing the SLM-to-objective magnification.

SLM models following the first strategy already exist, such as the Holoeye GAEA-2.1, with 4160 × 2464 pixels of 3.74 μm. If this creates larger steering angles, it also generates high dispersions as each wavelength of a laser pulse sees a slightly different phase and is reflected at different angles (Figure 3b, bottom panel). As shown in ref 97, for the bandwidths typically used in 2P optogenetics (central wavelength = 1030 nm, with a 1/*e* ^2^ spectral width < 30 nm), this greatly reduces the 2P efficiency at the sample plane and makes such a strategy practically nonviable. A possible solution could be to design specific optical elements that could compensate the SLM-induced dispersion, as discussed in ref 98.

The second strategy, i.e., using SLMs with a larger screen and larger pixels, as in ref 16, could be more suitable as it avoids undesired dispersion that can reduce 2P efficiency. Fabrication and cost-effective commercialization of similar SLMs is needed to deliver next-generation 2P holographic mesoscopes.

Another possible route to increase the accessible FOV is to couple the SLM with a scanning device that can produce larger angles, an idea already explored in ref 97, to produce quasi simultaneous patterning on very distant areas. In a different direction, tunable meta-surfaces^99,100^ were shown to produce large FOV holograms,^99^ and broadband meta-surface holograms were also demonstrated.^101^ New developments in this field could attempt to produce large-scale, tunable meta-surface SLMs with high throughputs (>80%), large steering angles (>±10°), and a wavelength-independent operation across typical laser bandwidth used in 2P optogenetic photostimulation experiments.

### Kilohertz 2P Voltage Imaging

Calcium imaging with GECIs is widely used for all-optical neurophysiology and it permits the activity of large numbers of neurons to be recorded simultaneously.^64,65,67,71,102,103^ Calcium transients generally last significantly longer (tens to hundreds of ms) than the underlying voltage fluctuations, facilitating the detection of neural activity (as they require slower imaging frame rates to achieve Nyquist sampling), but also limiting the quantification of spike firing rates and timing (the temporal resolution is limited by the duration of the calcium transient), particularly in the case of high-frequency trains of APs, as confirmed by simultaneous calcium and voltage imaging studies.^104^ Furthermore, GECIs are not well-suited for detecting subthreshold voltage changes (i.e., small changes in the membrane potential that do not cause neurons to fire) or hyperpolarisations resulting from synaptic and neuromodulatory inputs.^105^

Voltage indicators, which transduce changes in membrane potential into changes in optical signals, promise to address many of these challenges,^106^ and tremendous research efforts have been focused on developing bright and sensitive GEVIs.^29,95,107–110^ Detecting single APs requires millisecond recording precision, such that voltage indicators typically necessitate orders of magnitude faster imaging rates than with GECIs (kHz imaging rates). Similarly, the small signals associated with subthreshold postsynaptic changes in membrane potential (∼mV, with respect to ∼100 mV when an AP is fired) can only be detected using highly sensitive imaging approaches.

To overcome these challenges, optical voltage imaging experiments have generally relied on widefield, 1P illumination and camera detection, to maximize the number of fluorescence photons generated and collected.^111–113^ To approach single-cell resolution, background “crosstalk” from out-of-focus fluorescence has been reduced by using genetic approaches to achieve sparse labeling^114,115^ and/or targeted illumination based on amplitude or phase modulation.^29,116–120^

As already discussed, to further reduce out-of-focus fluorescence and reach deeper regions, the optical sectioning and longer excitation wavelengths inherent to multiphoton excitation microscopy can be exploited. However, the acquisition rate of conventional 2P-LSM is limited and millisecond transients, such as APs, can only be reliably detected by drastically reducing the FOV.^121–124^ More specialized imaging approaches, based on spatial and/or temporal multiplexing (in which the laser beam is divided into several beamlets that scan the sample at different spatial locations and/or with a relative delay of tens of nanoseconds) can record neural activity across much larger areas at kilohertz rates^93,109,125–129^ and can now operate close to the fundamental limit of fluorescence lifetime of the fluorescent proteins used (2–3 ns^130,131^ ).

Although classical or multiplexed approaches are valuable for imaging densely packed brain regions such as the hippocampus,^110^ neurons in other brain regions, and in particular, the cell membranes where GEVIs are expressed, generally occupy a small fraction of the total imaging volume, and make classical raster scan trajectories use the finite photon budget inefficiently.^129^

One approach capable of operating beyond this fundamental limit is to exclusively target regions of interest without spending time on dark or noninteresting regions of the sample. A subset of these techniques, random-access microscopy^91,92^ (RAMP), commonly uses acousto-optic devices in conjunction with holography to quickly (within ∼20 μs) scan the laser beam from one neuron to the next^94,95^ (Figure 3d). An exciting advancement of these methods demonstrated the use of high-speed 1D phase modulators to increase the point scanning rate of random-access microscopy by almost a factor of 7 (down to ∼ 3 μs).^132^ However, methods based on 1D phase modulation currently impose strict symmetry constraints on the output patterns,^133^ and the use of extended patterns to excite fluorescence at multiple points simultaneously results in the generation of out-of-focus fluorescence which can degrade signal-to-background ratio. Recently, deep 2P voltage imaging down to layers 5 and 6 of mouse cortex, was demonstrated at near-kHz frame rate, in a polygon-galvo scanning microscope combined with an adaptive excitation module,^134^ which selectively gates photon delivery to regions of interest during full FOV scanning. An alternative targeted voltage imaging approach is based on the combination of holography (using a SLM) with temporal focusing,^135,136^ and high-speed camera detection^96^ (Figure 3d). This method maintains single-cell resolution, even in scattering tissue, but further characterization of the fundamental imaging depth as compared with sequential approaches is necessary.

### Ultrafast 2P Optogenetics

Combining 2P CGH with advancements in fast-photocycle (few ms opening and closing kinetics), soma-targeted (i.e., whose expression is restricted to the neuron cell body) opsins, has permitted to elicit APs with submillisecond precision,^47,48,137–139^ and achieve high spiking rates up to 100 Hz,^138,139^ while keeping near single-cell spatial resolution. This precision and resolution allow the recreation of spatiotemporally precise activity patterns in single or multiple neurons in parallel, closely mimicking the physiological temporal dynamics observed in neural circuits.

However, replaying different temporal patterns in multiple cells on the ms level is challenging due to the relatively slow refresh rates of liquid crystal-based SLMs that can vary between ∼20 ms^43,48,137,139^ and 3 ms (or 2 ms in overdrive mode)^16^ for the fastest models, limited by the time required for liquid crystal molecules to rotate under specific voltages (Figure 3c).

Alternative technologies offer faster refresh rates but suffer from other limitations: ferroelectric SLMs^140^ can function at kHz refresh rates but only allow binary phase modulation to be generated; digital micromirror devices (DMDs)^141^ can typically reach >10 kHz but only in binary amplitude modulation, which strongly limits light efficiency in sparse targeting and therefore restricts usage to mainly 1P activation.^142–146^ Recently, MEMS (microelectromechanical system) technology offering both phase modulation and kHz switching rates in the visible spectrum has emerged,^147^ and while still in its early stages, future progress is likely to expand its use to the NIR.

Some groups have found workarounds, such as combining an SLM for generating illumination patterns with a DMD acting as a fast shutter for each spot,^90^ or tiling an SLM into independent phase masks accessible with an upstream scan unit at over 20 kHz^89^ (Figure 3c). These techniques require only the addition of a few optical elements (a DMD or a scanner, respectively) to holographic light shaping systems and could be easily implemented in laboratories already equipped with holographic technology. Increasing illumination reconfiguration speed has enabled the creation of submillisecond controlled temporal delays in the successive activation of two cells for the first time with 2P optogenetics,^89^ paving the way toward unrestricted playing of neural activation.

A real technological breakthrough for 2P optogenetics would be to develop systems capable of both mesoscopic holographic access (see Section Large-Scale Holographic Optogenetic Photostimulation) and ultrafast light-shaping reconfiguration, for large-scale and rapid neuronal activation. This might allow for instance to activate neurons in separated but connected brain regions to simulate or intentionally perturb the propagation of neuronal signals (on the ms scale) from one region to the other.

Ultrafast SLMs will not only be highly effective for inducing precisely timed neuronal activity through 2P photostimulation, but, as described in the next section, by taking advantage of the opsin photocycle, they can also help reduce the total power needed to quasi-simultaneously photostimulate large groups of neurons. On the 2P imaging side, techniques that are a mix of parallel and sequential RAMP approaches could be designed with ultrafast SLMs, in which a group of neurons is simultaneously excited before rapidly switching to a different group. Finally, in Part B, we will discuss adaptive optics and wavefront shaping approaches to counteract aberrations and scattering and thus, access deeper brain regions, which critically depend on the availability of rapid and stable SLMs.

### Scaling up Circuit Investigation within the Limits of the Sample

Achieving larger FOVs and faster acquisition speeds, or increasing the number of neurons targeted for simultaneous photostimulation (which can be necessary to induce detectable behavioral changes) often requires higher laser power, particularly in deep and scattering brain tissue, and can result in long and challenging acquisitions. In this section, we will first examine the potential risks of photodamage and photoinduced alterations to the sample, before discussing how leveraging specific properties of the sample can help minimize the required laser power, reduce artifacts, and maximize the overall efficiency and output of these experiments.

### Photoinduced Damages and Artifacts

Among the various types of laser-induced damage and undesired effects we can list thermal damages, nonlinear photodamage and artifactual neuronal responses.

Linear or thermal damages result from prolonged linear absorption of light; this is directly related to the amount of energy deposited on the sample, determined by the average power and duration of illumination. This heating is primarily driven by linear absorption of NIR light by biological tissues,^148^ especially water,^149^ and although heat is dissipated by blood flow, local or diffuse temperature increases can still occur. Even small temperature variations (<2 K) can influence various physiological aspects^150–154^ and larger increases (2–8 K) can lead to irreversible damage to the brain tissue, including protein denaturation and cell death.^155,156^ Temperature changes can disrupt the function of proteins such as opsins and other ionic channels, altering neuronal activity,^157–164^ and have broader implications like unintended behavioral alterations, such as body motion or changes in brain-wide neural dynamics.^165,166^ These effects can ultimately bias experiments. Interestingly, while brain heating is usually an undesired side effect, targeted photoheating of neurons expressing thermosensitive channels can be exploited to modulate neuronal activity, a technique known as thermogenetics.^167–169^

In standard 2P-LSM, using a conventional Ti:sapphire laser (<200 fs, 80 MHz), power levels typically range from a few to around 100 mW, and mouse brain studies have estimated a global brain temperature increase of about 2 K/100 mW.^148^ For optogenetic activation, low-repetition rate lasers (<300 fs, 0.5–10 MHz) with high pulse energy maximize 2P excitation while keeping the power per target down to a few mWs.^16,19,48,138,170^ During multicell (∼100 targets within a volume of a few hundred cubic microns) photostimulation, simulations predict a global rise of 1 K with localized “hot spots” of ∼2 K confined to a few micrometres and dissipating within milliseconds.^149^ Therefore, the heating threshold can get close to the operational ranges of both imaging and stimulation experiments and can pose a practical limit to the number of neurons that can be investigated. Carefully monitoring thermal effects, for instance, with experimentally validated temperature simulation models^149^ is essential, along with solutions for mitigating heating, as detailed in the following.

While high-peak power pulses can reduce photoheating, they may cause nonlinear damage due to the intense and localized electric fields they generate. These effects can result in photochemical damage, photoablation of tissues, optical breakdown, cell death, and the release of toxic substances into surrounding tissue, which can alter the overall behavior of the organism under study.^155,171^ Nonlinear damage has been observed at pulse energies in the range of 10 to 100s of pJ/μm^2^, sometimes only a few fold apart from the working power conditions of both 2P imaging and photostimulation.^48,89,172,173^ This concern becomes more significant in 3P excitation, which requires even higher peak power levels.

Finally, excitation light can elicit unwanted neuronal or behavioral responses by interacting with other photosensitive elements in the organism, such as photoreceptors in mammalian retinas, which can be activated by NIR light,^174^ or fish larvae that detect certain NIR wavelengths and exhibit light-avoidance behavior as a response.^175^

### Experiment Optimization Leveraging the Properties of the Sample

Specific properties of neurons in the brain, as well as those of activity indicators (GECIs and GEVIs) and opsins can be exploited to reduce laser power, minimize measurements, and shorten or simplify recordings. This not only reduces photodamage, but also improves experimental throughput and decreases data size to be stored and processed. We will now present practical examples that take advantage of the spatiotemporal sparsity of neural activity and connections, as well as the slow opsin photocycle. In addition to optimizing optical strategies, it is important to highlight that continuous developments in GECIs, GEVIs, and opsins^2,59,176^ are also essential for significantly reducing laser power requirements and minimizing photodamage.

### Leveraging Spatiotemporal Sparsity for Functional Imaging

As discussed earlier (Introduction and Section Kilohertz 2P Voltage Imaging), neurons in some brain regions are sparsely distributed, and their activity can be sparse in both space and time. This makes conventional point-scanning inefficient, as it often records from inactive regions, slowing down imaging speed and unnecessarily increasing data size. Spatiotemporal sparsity can be leveraged to optimize and speed up functional imaging using two key strategies:

(1) *Exploiting structural spatial sparsity* by restricting imaging to voxels of interest rather than raster scanning the full FOV. This reduces the number of measurements and thereby increases imaging speed and reduces data size, while also lowering energy deposition on the sample; RAMP techniques^91,92,177^ use this principle. However, its overall speed and signal-to-noise ratio (SNR) remain constrained by the sequential nature of target-by-target sampling.

(2) *Utilizing functional spatiotemporal sparsity* to allow reconstruction of individual activities from imaging where the full FOV is captured in fewer measurements using spatially extended point spread functions (PSFs). These parallelized sampling methods fall into two main categories: (i) scanning spatially extended point spread functions (PSFs) with single-pixel detectors (i.e., PMT) (reviewed in refs 30, 58, 178 ) or (ii) using scanless illumination with multipixel detectors (i.e., camera). In the first case, the scanned PSF can be an engineered single point (e.g., a Temporally-Focused low-NA Gaussian spot,^179^ a Bessel beam,^180^ a V-shaped PSF^181^), a line,^93^ or multibeam illumination (such as multiplexed sets of points distributed in 3D for multiplane imaging^98^). A drawback of the approaches above is their reduced spatial resolution and increased crosstalk between emitters, simultaneously recorded by the single detector. In scanless imaging,^26,96,182,183^ instead, the camera preserves spatial information about emitters. However, as imaging depth increases, scattering progressively disrupts this spatial encoding and leads to signal loss.

In both cases, demixing algorithms^184–186^ can help mitigate these issues by using known fluorescent dynamics of activity reporters and brain activity sparsity to separate overlapping signals. Such approaches have successfully reconstructed fluorescent transients from highly intermixed signals, like scattering patterns through multimode fibers^187^ or diffusing tissues.^188^ Interestingly, the combination of dynamic illumination patterns and temporal sparsity may further improve demixing performances.^116,189,190^ Moreover, as the field transitions from calcium to voltage imaging—where fluorescent transients are faster and more temporally intertwined—these demixing methods will become even more critical.

### Leveraging Sparse Connections and Functions for Circuit Photostimulation

2P optogenetic photostimulation can be used to selectively activate or silence even one individual neuron within an ensemble. While cell-by-cell photostimulation can help identify neuronal functions and map synaptic connections, scaling this approach to larger circuits (involving thousands of neurons) remains challenging and often inefficient, as it requires many measurements.

A key example is synaptic connectivity mapping. Neurons communicate through synapses, where activation of a presynaptic neuron generates an electrical signal in the postsynaptic neuron if they are connected. Sequentially photostimulating (one-by-one) hundreds of potential presynaptic cells while recording (with an electrode) from a single postsynaptic neuron can partially map the functional connectivity of local circuits.^47,49,191–196^ Yet, this method is inefficient due to the sparse nature of synaptic connections, leading to many photostimulations that fail to evoke a response in the postsynaptic cell.

To overcome this inefficiency, similar to parallelized imaging, group testing approaches have been proposed.^197–199^ Instead of stimulating neurons one by one, multiple neurons are activated simultaneously to integrate their inputs on the postsynaptic neuron. This approach, recently validated experimentally,^194,196,200^ combined with signal reconstruction techniques, is an example of compressed sensing^201^ and can more efficiently probe the full population. By reducing the number of required stimulations, this approach enables faster network mapping with fewer recordings, making it also particularly useful when the effects of single-cell activation are subtle or challenging to detect. As pioneering studies of visual encoding,^15^ locomotion,^21^ and sensory modulation^12,18–20^ have demonstrated, precise multicell photostimulation will likely become a standard tool in Neuroscience.

### Exploiting the Opsins Photocycle for Power-Efficient Multicell Photostimulation

Unlike fluorophores, which have nanosecond relaxation times, opsins exhibit a slower photocycle, staying open for several milliseconds after excitation. Leveraging this characteristic, along with the ability to generate rapid holographic stimulation patterns (see Section Ultrafast 2P Optogenetics), a novel cyclic illumination protocol has been developed to maximize the number of activated cells within a fixed power budget.^89^ Instead of splitting the laser power across all targeted neurons, subgroups are activated sequentially and the illumination is delivered in short bursts (50 μs) to each subgroup. Due to their slow relaxation, opsins in the first subgroup remain active while the laser moves to the others. By cycling back and repeating this process, all neurons can spike nearly simultaneously, while the overall power consumption is significantly reduced compared to simultaneously targeting the entire neuronal ensemble.^89^

To conclude Part A, we have discussed challenges and perspectives for increasing the FOV and speed of photostimulation and imaging. A clear path for future developments is to merge together the efforts toward larger FOV and faster performances, both for 2P imaging and 2P photostimulation, which often advance in parallel without being implemented in the same platform. As we have seen in this section, the very nature of the sample under study will have to be considered to design specific strategies that minimize tissue damage and optimize recordings.

## Part B. Accessing deep brain regions

Brain tissue challenges light propagation due to absorption and scattering. With increasing depth *z* within the tissue, the light intensity *I*(*z*) decreases exponentially:^31,202^

$$I\left(z\right)\propto e^{-z/l_{e}}$$

where *l*_e_ is the extinction or effective attenuation length, defined as $l_{e}=l_{\mathrm{abs}}+l_{s}$ , with $l_{\mathrm{abs}}$ and $l_{s}$ representing the absorption and scattering length, respectively. Absorption in biological tissues is mainly due to water, but for wavelengths shorter than ∼1400 nm, the extinction length is primarily dominated by scattering.

Compared to the visible spectrum, longer, NIR wavelengths experience less scattering (140 μm < $l_{s}$ < 260 μm for 800 nm < λ < 1100 nm compared to $l_{s}$ < 110 μm for λ < 700 nm^203^) and are thus beneficial for deeper all-optical experiments. This advantage can be exploited by using red-shifted calcium indicators or optogenetic actuators (opsins), though spectral cross-talk between the two must be considered^48,204,205^ to be combined in all-optical experiments. Switching to 2P excitation, which uses NIR lasers and provides increased axial confinement, is often the best choice for precise all-optical experiments in the scattering mouse brain.^31^

However, the depth at which 2P microscopes can effectively image is constrained by a loss of contrast linked to the appearance of out-of-focus fluorescence as the excitation power is increased to compensate for depth loss.^206–208^ For the mouse brain, this generally limits 2P functional imaging to 0.6–0.7 mm below the surface with green calcium indicators^209–212^ and slightly deeper, up to 0.85 mm with red-shifted calcium reporters,^213^ overall limiting experiments to the upper layers of the cortex.

To reach deeper brain regions, different and complementary strategies have been pursued: (1) transitioning toward even longer excitation wavelengths by switching to a three-photon (3P) excitation regime; (2) correcting scattering-induced wavefront deformations with adaptive optics and wavefront shaping; (3) implanting minimally invasive devices to relay light from the surface to the deep brain regions of interest.

### Three-Photon Excitation Microscopy

Three-photon excitation microscopy (reviewed here;^214,215^ Figure 4a) enables deeper brain access by using longer wavelengths for excitation and by further reducing out-of-focus excitation thanks to its higher nonlinearity. In the NIR range, wavelengths around 1300 and 1700 nm offer optimal scattering/absorption trade-offs ( $l_{e}$ ≈ 250–300 μm at 1300 nm; $l_{e}$ ≈ 400 μm at 1700 nm),^203,216,217^ and also align with the tripled frequencies of green and red-emitting fluorophores.

**Figure 4:**
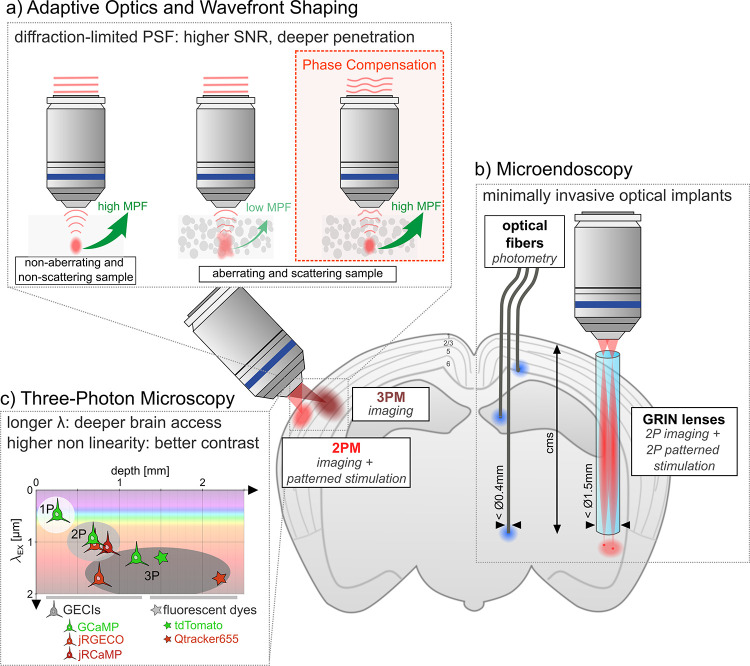
Strategies for deeper brain access. (a) Adaptive Optics. In the absence of aberrations, a diffraction-limited focal spot is formed in a point-scanning microscope, leading to high multiphoton fluorescence (MPF) generation. Inside a scattering medium such as brain tissue, mismatches in the refractive index distort the wavefront, resulting in a distorted, enlarged focus and reduced MPF. Preshaping the wavefront to compensate for tissue aberrations allows for the recovery of a tight focus and high MPF. (b) Micro-Endoscopy. Minimally invasive optical implants enable optical access to brain regions at centimeter depths. Thin optical fibers (<Ø 0.4 mm) can record fluorescence from a specific region (1P photometry) and are multiplexable. GRIN lenses (<Ø 1.5 mm) can be coupled to a 2P all-optical system to perform 2P point-scanning imaging and 2P patterned optogenetic stimulation. (c) Three-Photon Excitation Microscopy. Longer NIR wavelengths and higher-order nonlinearity help maintain imaging contrast at millimeter depths. Maximum recorded imaging depths for GECIs under 1P, 2P, and 3P excitation and different excitation wavelengths are shown. For comparison, maximum imaging depths of fluorescent dyes and quantum dots under 3PE are also represented.

Due to the low 3P action cross sections of fluorophores,^218^ 3P imaging has only been possible thanks to the development of low-duty cycle amplified lasers (∼1 MHz, sub-100 fs, pulse energies of few μJ) operating in the 1300/1700 nm spectral regions.^203^ In vivo 3P calcium imaging using 1300 nm excitation of GCaMP has been demonstrated^219^ and widely adopted,^211,220–222^ notably allowing imaging through all cortical layers and reaching the hippocampus at a 1 mm depth in the mouse brain. Similarly, 1700 nm excitation of red-shifted GECIs (jRGECOs)^129^ has enabled deep cortical imaging in the mouse brain (up to z = 0.75 mm). However, despite improvements,^213^ red-shifted indicators remain several times dimmer than GCaMP, which limits their maximal fluorescence recording depth.

Unlike 2P imaging, which is limited by contrast loss at depth, 3P imaging maintains minimal background noise at greater depths due to its higher order of nonlinearity,^216,223,224^ but is rather limited by challenges in experimentally generating and/or collecting sufficient signal, and/or tissue heating.^178^ Theoretically, 3P microscopy at 1300 and 1700 nm could maintain contrast up to 9 extinction lengths ( $l_{e}$ ;^203,225^ approximately 2.5–3.5 mm depth). Experimentally, however, maximum reported imaging depths are typically around 5 $l_{e}$ ^226,227^ with bright dyes or quantum dots, and drop to about 4 $l_{e}$ ,^211,222,228^ and 2 $l_{e}$ ^129^ with green and red GECIs, respectively, owing to their lower brightness.

Enhancing the brightness of GECIs or developing functionalized quantum dots for calcium sensing^226^ could extend 3P calcium imaging depths within the 5 $l_{e}$ limit achieved by dyes and quantum dots. Complementary strategies to enhance fluorescence generation include optimizing system transmittance (with customized optics,^203^ appropriate immersion media,^229^ underfilling the pupil of high NA objectives^230^) and employing adaptive optics.^231–234^ Photon budget can also be optimized by using Adaptive Excitation Sources (AES),^129^ which synchronizes laser pulse delivery with pixels of interest during raster scanning of the FOV. AES has been demonstrated to keep average powers (and hence temperature rises) low. This approach holds significant potential for addressing both heating challenges and the low imaging frame rates in raster scanning configurations, which are limited by the ∼1 MHz repetition rates of laser sources in 3P microscopy. On the fluorescence detection side, there is great potential for improving photon collection efficiency, with an estimated 3-fold margin for enhancement.^235^ Strategies for optimizing detection efficiency include using low-magnification high-NA objective lenses^236,237^ and specialized PMTs^238^ designed for 3P microscopy.

As of today, 3P microscopy is primarily used for imaging of neurons and neuronal activity. Parallel developments aimed at reaching greater depths for optogenetic photostimulation may in the future extend all-optical experiments to the deepest regions of the mouse cortex. However, 3P optogenetics photostimulation^239^ might be limited by the higher energy demands of optogenetic activation compared to point-scanning imaging. Such experiments could benefit from the development of lasers with higher deliverable energies.

### Adaptive Optics, Wavefront Shaping

Light focusing within deep brain tissues is degraded by optical aberrations and scattering, both caused by refractive index and surface inhomogeneities (see Figure 4a). These effects broaden the excitation PSF, resulting not only in reduced spatial resolution but also in decreased focal intensity, and consequently diminished fluorescent signal in multiphoton microscopy, particularly for higher-order processes.^240^ Compensating for this focal intensity loss by increasing power can heighten the risks of linear and nonlinear damage, while the enlarged PSF can also lead to signal contamination from structures outside the diffraction-limited spot during deep calcium imaging experiments.^241,242^ Although scattering and optical aberrations both impact focus, resolution, and contrast, they differ in their compensation challenges in biological tissue. Optical aberrations arise from smooth, low-frequency refractive index variations over larger spatial scales, typically caused by structures larger than the wavelength of the NIR laser, such as brain layers or blood vessels,^243^ and can be corrected using adaptive optics. In contrast, scattering results from high-frequency, fast variations in the refractive index caused by structures comparable in size to the wavelength of light (e.g., cells, nuclei, organelles etc.).^243^ This leads to a speckle pattern, i.e., a deterministic interference pattern from randomly distributed coherent sources, that is much harder to compensate for using traditional correction methods and that poses a significant challenge in today’s research.

### Compensating Optical Aberrations: Adaptive Optics

Originally developed for astronomy,^244^ Adaptive Optics (AO) has proven highly effective in correcting aberrations in multiphoton microscopy, resulting in enhanced contrast and diffraction-limited imaging at increased depths (typ. maximum 0.7 mm in 2P,^245^ 1.2 mm in 3P^231–234^ ). While large structures (e.g., neuronal bodies ∼10 μm) partially compensate for the reduced intensity caused by PSF enlargement due to the excitation of more fluorophores, this effect critically limits the imaging depth for smaller structures^242,246^ (e.g., dendrites, spines). AO operates by measuring aberrations and dynamically compensating for them using a wavefront corrector, such as an SLM or a deformable mirror. Over the past few years, numerous AO implementations have been proposed and applied to brain imaging (see reviews in refs 244, 247, 248 ). They can be broadly categorized depending on whether they rely on direct or indirect wavefront sensing techniques. Briefly, direct methods typically employ a Shack–Hartmann wavefront sensor to measure in a single shot the distorted wavefront originating from a spatially coherent “guide star”, e.g., formed by the 2P excitation of a red-shifted dye.^242,245,249–251^ Indirect methods, a.k.a. “sensorless” methods, estimate the aberrated wavefront by sequentially adjusting the pattern on the wavefront corrector to maximize a focusing metric (e.g by monitoring 2P fluorescence signals) by means of an optimization generally performed through a zonal (e.g., pupil segmentation^252,253^ ) or a modal approach (e.g., Zernike modes scan^254,255^ ).

Direct methods are fast, provide high measurement accuracy for optimal correction, and can be used with time-varying fluorescent signals, such as those in calcium imaging.^256^ In contrast, indirect methods do not often require guide stars, are more robust to scattering, and are simpler to implement since they only require a wavefront corrector. Moreover, the recent development of wavefront correctors that work in transmission^257,258^ is expected to further promote the dissemination of AO in Neurophotonics. These devices eliminate the need for additional beam-folding optics and can be directly placed at the pupil of the microscope objective, making integration easier. Although these correctors are relatively slow (typically few ms) and limited in the number of modes they can address, they are sufficient for correcting low-order aberrations that can be considered as quasi-static in microscopy and can be described typically by 7–11 Zernike radial orders.^244^

### Compensating Scattering: Wavefront Shaping

However, it should be emphasized that the correction of smooth, low-order aberrations can only enhance ballistic contributions. Yet, at depths higher than the scattering length $l_{s}$ (typ. $l_{s}$ = 150 μm at λ_2P_, and $l_{s}$ = 300–400 μm at λ_3P_ in the brain), scattered light becomes predominant. It typically accounts for more than 99% of the deposited energy at depths beyond 5 $l_{s}$ . Over the past decade, significant advances have been made in the development of techniques for understanding and controlling light propagation in complex media (see reviews^202,259^ ). In particular, “wavefront shaping” holds the promise of harnessing scattered photons to enable deep imaging in brain tissues, overcoming the limitations imposed by the exponential attenuation of ballistic photons with increasing depth. It involves not only compensating for low-order aberration as in conventional AO, but also correcting the high-order wavefront distortions, mainly composed of optical vortices,^260^ induced by multiple scattering. Performing such phase correction^261^ using a nonlinear optical feedback^262^ is particularly challenging since (i) it requires probing/controlling wavefront distortions that involve thousands to millions of spatial modes to focus light. Additionally, (ii) to reach optimal performances, the phase compensation must be carried out faster than the speckle decorrelation time which corresponds to the time at which the wavefront loses correlation with its earlier state (typ. below ∼1 ms at millimeters depth in living mouse brain^263^). Note that correcting more persistent modes is still possible but comes at the cost of reduced signal enhancement.^264^ Finally, (iii) the scattering compensation is only effective over a limited FOV determined by the angular memory effect range, which is related to the intrinsic isoplanatism of the scattering process (typ. from tens of μm at shallow depth to the size of a speckle grain at large depth). Performing imaging through scattering media on a large FOV ideally requires estimating the so-called “Transmission Matrix” (TM), which linearly relates the input field to the output field.^265,266^ A recent study^267^ has demonstrated the possibility of measuring the TM using a 2P signal, a computational framework, and a single-pixel detector (e.g., a PMT). This represents a significant advancement over traditional camera-based strategies, which generally lack the sensitivity for deep imaging in a strong multiple scattering regime.

Over the past few years, significant efforts have been made to address this challenge in the context of multiphoton brain imaging.^259^ Briefly, indirect, iterative, interferometric methods such as IMPACT,^268^ F-SHARP,^269^ α-FSS,^234^ and DASH^270^ have already demonstrated impressive 2P and 3P signal enhancement (typ. 1–2 orders of magnitude) *in vivo*, but mainly at moderate depth (i.e., in an intermediate scattering regime) in the brain tissue or through the intact skull (i.e., through a static scattering medium). Moreover, conjugated^271^ and multipupil^272^ correcting strategies have proven relatively efficient at extending the corrected FOV to a certain extent, but innovative solutions are required for larger-scale correction.

Considering the giant number of TM modes that must be probed for large-scale correction in the multiple scattering regime, strategies based on random access, which exploit neuronal sparsity, should be prioritized, as they minimize the number of isoplanatic patches requiring measurement and correction. A recent approach^273^ combining fast (40 kHz) AOD-based wavefront shaping with 3D random-access scanning has shown great promise for providing fast, iterative multipatch correction in transcranial imaging. However, despite important speed improvement, these indirect methods remain iterative, requiring multiple SLM updates for each patch, keeping them well below the potential performance limits of current SLM refresh rates. In contrast, a single-shot “digital optical phase conjugation” method has been recently demonstrated in model sample using 1P fluorescence guide stars,^260^ by exploiting the large spectral bandwidth of forward multiple-scattering media^274^ along with a high-resolution wavefront sensor capable of reconstructing speckle fields.^275^ Extending this approach to two-photon guide stars and coupling it with multiplexed wavefront sensor schemes^276^ presents an intriguing prospect for probing multiple isoplanatic patches in a single-shot.

Across these strategies, the availability of an ultrafast SLM^277^ (typ. 0.1–1 MHz) with a high number of modes and working in the NIR would be a true game changer, significantly accelerating measurement in feedback-based methods and/or speeding up compensation in various correction strategies.

### Minimally Invasive Implants: GRIN Lenses and Fibers and Wavefront Shaping

Many behaviorally relevant brain structures lie below 1 mm inside the brain,^278^ beyond the current reach of optimized (3P, AO) microscopes. Presently, the only viable approach to get optical access to them is the implantation of a relay system (Figure 4c) that has to be minimally invasive, while ensuring access to a sufficiently large FOV and to high quality optical recordings. There are two main families of widely used relays: gradient refractive index (GRIN) lenses, and optical fibers (Figure 4b).

GRIN lenses are small cylinders of glass (diameter < 1.5 mm, several mm long), which serve as imaging relays.^279,280^ Their main drawback is their large optical aberrations that eventually limit the accessible FOV to a size smaller than the GRIN lens diameter. Proposed solutions include adaptive optics^281–283^ and correction lenses at the GRIN lens entrance.^284,285^ Commercial GRIN lenses (for instance from Grintech, ref.^286^) can offer field and chromatic corrections, at the cost of larger diameters and increased invasiveness. GRIN lenses have been used for 2P all-optical experiments with CGH in both superficial^287^ and deep^14,288^ brain regions. Advanced techniques have increased the available FOV, through using multiple GRIN lenses, coupled to a single objective, to simultaneously study several brain regions,^73^ or through a GRIN lens-microprism assembly that give access to full panoramic views of an entire brain column.^289^ Microprism assemblies, recently extended for deeper brain access,^290,291^ offer a lower-aberration alternative to GRIN lenses.

Using optical fibers to guide the light has the advantage of being truly minimally invasive, as typical fiber diameters are in the range of 50–400 μm.^278,292^ Fiber photometry experiments, in which all the cells expressing an activity reporter are simultaneously excited and detected through the fiber,^278,292^ or fiber optic widefield 1P optogenetic photostimulation are a relatively common technique in Neuroscience. These experiments can be improved by using multiple fibers^293^ and by engineering the fiber tip and exploiting plasmonic effects to enable optical access to different points within the same fiber.^294,295^

By using SLMs and wavefront shaping,^202^ it is possible to turn a multimode optical fiber (MMF) into an imaging device,^296,297^ which has recently allowed researchers to record morphological images and neuronal activity in very deep brain structures.^298^ In this case, wavefront shaping techniques, similar to the ones described above in the context of scattering, allow researchers to focus and scan a laser beam through the MMF to construct an image. In the future, shaping two different lasers with separate SLMs could allow simultaneous imaging and photostimulation.

A limitation of these approaches is their sensitivity to fiber bending and deformations, which has so far limited their use to static fiber conditions. As we will detail in the next section, optical fibers could be the ideal platform for both deep brain (thanks to their small footprint) and studies in freely moving animals (exploiting their flexibility). However, wavefront shaping through fibers in dynamic conditions has so far remained elusive. As different groups are currently tackling this problem,^299–302^ we expect that powerful solutions will emerge in the near future.

## Part C. Freely Moving Mice

The all-optical techniques described so far are restricted to the use of head-restrained animals, which limits behavioral studies. Understanding how neuronal circuits affect certain types of behavior, necessarily calls for new methods that should be capable of studying animals engaged in naturalistic behavioral tasks. Recent efforts focus on developing lightweight (<5g) miniaturized wearable systems for optically studying neuronal activity in freely moving rodents (for comprehensive reviews, see ref 303–305 ). Many of those systems have been only used for calcium imaging, with few notable example of all-optical experiments.^24,25,306^ Miniaturized optical systems fall in 3 main families (Figure 5).

**Figure 5:**
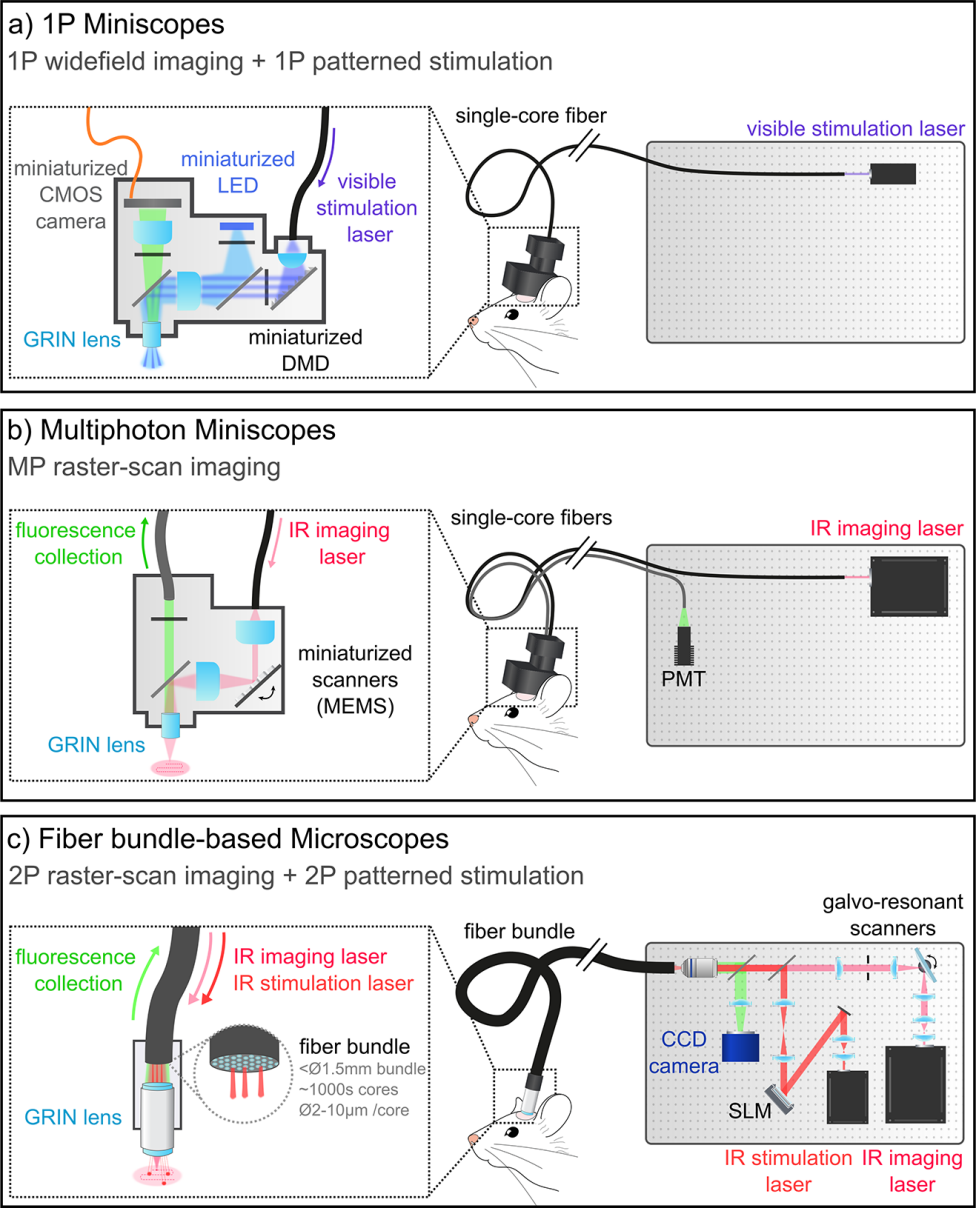
Optical systems for all-optical experiments in freely moving mice. This figure illustrates miniaturized microscopes (center), with a detailed view of the optical components on the animal’s head (left) and those remaining on the optical table (right). (a) 1P miniscopes for functional imaging are fully mounted on the animal’s head and include miniaturized LEDs and CMOS cameras. Some systems incorporate 1P patterned illumination (1P MAPSI^25^) for optogenetic stimulation by integrating a Digital Micromirror Device (DMD) and relaying a visible laser from the optical table to the miniscope using a single-core fiber. (b) In 2P miniaturized microscopes, the NIR imaging laser is delivered via fiber to miniaturized scanners (MEMS) on the miniscope for 2P point-scan imaging. Fluorescence is collected through another fiber and detected by a PMT. Currently, 2P miniaturized microscopes do not support 2P patterned optogenetic stimulation. (c) In fiber bundle-based microscopes, standard optical systems for 2P point scanning (galvo-resonant scanners) and 2P patterned optogenetic stimulation (SLM) are located on the optical table. Both lasers are relayed to a GRIN lens on the animal’s head via a fiber bundle, which also collects fluorescence.

### 1P Miniscopes

1P miniscopes are complete microscopes that fit on the animal’s head, featuring miniaturized LEDs and CMOS cameras. They share the aforementioned advantages and drawbacks of 1P microscopes: they are a cost-effective solution, offer large (up to 5–10 mm) FOVs^75,307,308^ at high acquisition rates (up to 500 Hz^309^), but are limited in penetration depth and affected by high fluorescence background due to poor axial sectioning. To extend miniscopes to all-optical studies, a new system was proposed,^25^ utilizing an optical fiber to transmit visible laser light from the optical table to the animal head and a miniaturized DMD for patterned optogenetic photostimulation with near single-neuron resolution. 1P miniscopes are already routinely used in combination with GRIN lenses to reach deep brain regions in freely moving mice. To minimize invasiveness, it was recently proposed^187^ to implant a short (8 cm) optical fiber instead of a GRIN lens and detect the fluorescence form GECIs backpropagating through the fiber with the miniscope camera. By using non-negative matrix factorization algorithms, it could be possible to temporally demix neuronal activity traces as each neuron produces a recognizable spatial fingerprint on the camera. Going a step further, and making use of a miniaturized DMD,^25^ in the future it might become possible to perform wavefront shaping directly at the animal head through the same short optical fiber to focus a visible laser beam to the locations of choice. In this way one could at the same time record demixed calcium activity traces and photostimulate neurons with the DMD, all in a compact and cost-effective device, with the advantage that with short fibers almost completely implanted, the risk of wavefront distortions due to fiber bending could be minimized. Another innovation in portable, real-time, large FOV imaging is the 1P masked-based lens-less miniscope technique,^310^ which substitutes bulk optics with a thin optical mask and additionally provides scanless volumetric access.

### Miniaturized Multiphoton Microscopes

Miniaturized multiphoton microscopes utilize single-core optical fibers to transmit the NIR laser beam from the optical table to a head-mounted microscope equipped with miniaturized optics and 2D scanners (mostly MEMS,^222,311^ but also fiber scanner^312^); fluorescence is also fiber-collected and detected by synchronized PMTs. They offer high-resolution 2P^311–313^ and 3P^228,314^ calcium imaging with FOVs up to ∼1 mm^313^ and frame rates <50 Hz, and potential z-scanning using miniaturized tunable lenses.^311^ A key limitation of these devices is that they have not yet been coupled to 2P optogenetic photostimulation. A possible solution could be to develop a miniaturized phase-only SLM, for instance based on reconfigurable thermo-optics^315–317^ or metasurfaces^318^ to be placed directly at the animal head after the fiber. Most SLMs usually operate in reflection mode, such that integrating them into an optical system demands the optical path to be folded, thus increasing weight and volume. Thermo-optics and metasurfaces instead, can operate in transmission, which is very appealing to develop highly compact and low-weight devices.

### Fiber Bundle-Based Microscopes

Fiber bundle-based microscopes utilize multicore fibers, also known as fiber bundles, consisting of thousands of individual cores (diameter of 2–10 μm), enabling them to function as imaging systems. They relay optical signals between the optical table and a miniaturized objective (e.g., GRIN lens) on the animal’s head. Typical elements for 2D imaging (standard galvo scanners) and patterned optogenetic activation (SLMs) remain on the optical table (except for z-scanning tunable lenses^319,320^ ) and do not require miniaturization. The accessible FOV depends on the bundle’s diameter (up to 1.5 mm) and the optical system’s magnification at the fiber output. These microscopes support both 1P^24,321,322^ and 2P^306,319,320^ imaging, as well as 2P CGH using an SLM before the fiber that has recently demonstrated optogenetic activation with single-cell precision in freely moving mice.^306^

We expect performance to greatly benefit from novel bundle architectures, such as bundles with a larger diameter and higher core number, which must still be flexible enough to allow animals to freely move. In this sense, leached fiber bundles, which lack a shared clad between all the cores, could be a potential solution to maintain flexibility, but to our knowledge they are only produced in standard lengths/diameters by two manufacturers (Schott, Sumita), with little or no customization available. Finally, similar to what described in the previous section for multimode fibers, wavefront shaping methods through fiber bundles are also capable of focusing laser light with no additional micro-optics.^302,323,324^ At the same time, imaging through fiber bundles using holographic detection and computational reconstruction methods are also being extensively investigated.^300,325,326^ Hybrid approaches that leverage on wavefront shaping and computational methods to enhance existing fiber bundle-based microscopes could be a way to explore in the future.

It is foreseeable that research toward freely moving studies will in the future merge with that of minimally invasive components to reach deeper brain regions (Section Minimally Invasive Implants: GRIN Lenses and Fibers and Wavefront Shaping). In order to rapidly advance with both, it is necessary to invest in the broad availability and improvement of miniaturized components. High resolution 3D printing is a promising way to fabricate aberration corrected miniaturized optics, even on the tip of optical fibers, but are so far restricted to only a handful of research groups.^327–332^ Tunable metasurfaces^99,100^ designed from the beginning for neurophotonics applications might provide extremely valuable components to best study neuronal circuits in freely moving animals.

## Conclusions

All-optical systems that combine the recording of neuronal activity with controlled optogenetic perturbation offer highly promising methodologies for neuroscience research in small animal models. As optical techniques rapidly evolve, this manuscript has focused on highlighting emerging advancements and required improvements in key technological components and approaches. While this Review has not primarily focused on the development of indicators or actuators, it is important to emphasize that optics alone will not be sufficient to address all the challenges faced by neurophotonics. Advancing the field will require the development of more sensitive and soma-targeted opsins, brighter and more reliable indicators for calcium and voltage, and the minimization of spectral crosstalk between opsins and indicators. These represent critical areas for improvement that will have a significant impact on the future of Neurophotonics. In what follows, we summarize some of the key technological advancements and challenges discussed in this manuscript:

### Commercialization of State-of-the-Art Techniques

The commercialization of large and advanced technologies developed in specialized laboratories is critical for broader adoption in research environments with strong biology and neuroscience expertise but less familiarity with complex optics. While 2P all-optical microscopes are becoming more accessible, new key technologies require either all-in-one solutions or modular components that can be integrated into existing systems. In microscopy, the commercialization and widespread adoption of 2P mesoscopes for ultrawide calcium imaging and kHz imaging modules would mark a significant advance. A breakthrough solution would be a multifunctional microscope capable of seamlessly handling large FOV, kHz speeds, 2P–3P imaging, and freely moving configurations. On the laser side, current offerings meet key needs with powerful Ytterbium-doped laser amplifiers for multicell 2P optogenetic activation and optical parametric amplifiers (OPAs) for 3P microscopy. However, commercialization of AES (Adaptive Excitation Source) technology as an integrable module for deep 3P imaging would further extend-capabilities, and stronger 3P laser sources may still be required for deep all-optical experiments. Tunable high-power lasers, such as OPAs, that cover the full wavelength range used in 2P optogenetics (approximately 800–1200 nm) could improve spectral opsin matching,^37^ thereby reducing power demands and minimizing heat generation during multicell stimulation.

### Optimization of SLMs and off-the-Shelf Optical Components

As we have seen throughout this manuscript, phase-only SLMs are likely one of the most critical optical elements for neurophotonics. Researchers need larger-screen SLMs with more pixels to extend FOE for optogenetic photostimulation and higher-speed SLMs (>kHz) for naturalistic activity replay, random access microscopy, scattering correction and compressed sensing strategies. Miniaturized SLMs working in transmission using thermo-optics or metasurfaces would enable compact systems for freely moving animal experiments. It is also key to optimize the performance, accessibility and customization possibilities of standard optical components (e.g., specialized PMTs and objectives for 3PM, low-aberration GRIN lenses), and miniaturized optical components (e.g., MEMS scanners, DMDs, tunable lenses, filters, detectors). Miniaturized custom optics can also be produced using advanced 3D printers, such as Nanoscribe;^330,331^ however, these printers are currently prohibitively expensive, making it inefficient for each lab to own one. Therefore, establishing more companies that offer custom design services would significantly enhance accessibility. Fiber optics are increasingly used in microscopy and offer crucial advancements for multiregion imaging, deep tissue imaging, and flexible systems for freely moving experiments with maintained precision, as well as multicolor light patterning. For further progress, research laboratories require the development of custom fiber such as large-diameter bundles with more cores, bend-resilient fibers for wavefront shaping, and higher NA multimode fibers for improved signal collection.

### Advancements in Software and Data Methodologies

Advancements in software and signal reconstruction algorithms are essential for maximizing the impact of all-optical experiments. Demixing algorithms could revive the use of 1P techniques in mini-, micro- and meso-scopes, particularly for large-field, fast imaging (e.g., voltage imaging); and extend the depth limits of standard multiphoton and fiber-bundle-based microscopes. As data volumes grow, developing robust pipelines for processing large data sets will be critical. Equally important is providing open access to this data, enabling other research groups to analyze it or use it to create computational models, fostering greater collaboration and accelerating discoveries. Additionally, integrated software and hardware solutions to implement fast and optimized multicell stimulation protocols will facilitate large-scale network analysis with minimal data and reduced photodamage.

We believe that advancements in these areas will be crucial for driving the next wave of progress in Neurophotonics. These innovations will not only enhance the precision and capabilities of optical techniques but also spur new discoveries in Neuroscience, ultimately deepening our understanding of brain function. They will enable establishing precise correlations between brain activity and complex behaviors (e.g., social interactions or motor behaviors) in natural, freely moving conditions. Moreover, advancements in microendoscopy, adaptive optics, and multiphoton microscopy will allow exploration of deep brain structures involved in higher-order cognitive functions like memory and decision-making. High-speed recordings, in particular the emerging field of voltage imaging, along with millisecond-precise optogenetic manipulation, could help resolve the long-standing debate on whether information in the brain is temporally encoded in the form of spike timing or spike frequency. Finally, large-field-of-view imaging will enable full-brain or even full-animal studies in small organisms (like C. elegans or zebrafish), shedding light on long-range connectivity and inter-regional communication.

## #

Brain Research Institute, University of Zurich, Zurich, Switzerland
